# 837. Isavuconazole in the Treatment of Chronic Forms of Coccidioidomycosis

**DOI:** 10.1093/ofid/ofad500.882

**Published:** 2023-11-27

**Authors:** Arash Heidari, Rupam Sharma, Qusai Shakir, Madiha Shah, Monica Donnelly, Trina Reynolds, Kate Trigg, Jeff Joliff, Rasha Kuran, Royce H Johnson, George R Thompson

**Affiliations:** Dignity Health, Bakersfield, CA; Kern Medical Center, Bakersfield, California; Kern Medical Center, Bakersfield, California; University of California Davis Medical Center, Sacramento, California; University of California Davis Medical Center, Sacramento, California; University of California Davis Medical Center, Sacramento, California; University of California Davis Medical Center, Sacramento, California; Kern Medical Center, Bakersfield, California; Kern Medical Center, Bakersfield, California; Kern Medical Center, Bakersfield, California; University of California Davis Medical Center, Sacramento, California

## Abstract

**Background:**

Coccidioidomycosis is a fungal infection with a range of clinical manifestations. Currently used antifungal agents exhibit variable efficacy and toxicity profiles necessitating evaluation of additional therapeutic options.

**Methods:**

Patients with coccidioidomycosis who received Isavuconazole were identified by cross-indexing ICD-9 and ICD-10 codes from patients and data abstracted. Responses to Isavuconazole therapy were measured using a modified Mycoses Study Group Coccidioidomycosis Scoring system as described previously.

**Results:**

82 patients met the criteria for inclusion. Over half of the patient exhibited pulmonary involvement 45/82 (55%), although meningitis 32/82 (39%), bone and joint disease 14/82 (17%) and skin/soft tissue infection 7/82 (9%) were also seen. The majority of patients experienced a decrease in their MSG score following initiation of Isavuconazole therapy (median MSG score change across all patient groups 7 → 2, p< 0.0001). Overall improvement was noted in 58/82 (71%) patients, while no change was observed in 19/82 (23%) and 5/82 (6%) who were unresponsive to antifungal changes.

**Conclusion:**

Isavuconazole demonstrated efficacy in the majority of patients treated, with failures observed only in a subgroup of patients with coccidioidal meningitis.

**Disclosures:**

**Rupam Sharma, PGY-1/MD**, Astellas: Grant/Research Support **George R. Thompson, III, MD**, Astellas: Advisor/Consultant|Astellas: Grant/Research Support|Cidara: Advisor/Consultant|Cidara: Grant/Research Support|F2G: Advisor/Consultant|F2G: Grant/Research Support|Mayne: Advisor/Consultant|Mayne: Grant/Research Support|Melinta: Advisor/Consultant|Melinta: Grant/Research Support|Mundipharma: Advisor/Consultant|Mundipharma: Grant/Research Support|Pfizer: Advisor/Consultant|Pfizer: Grant/Research Support

